# Event-Related Desynchronization Induced by Tactile Imagery: an EEG Study

**DOI:** 10.1523/ENEURO.0455-22.2023

**Published:** 2023-06-14

**Authors:** Lev Yakovlev, Nikolay Syrov, Andrei Miroshnikov, Mikhail Lebedev, Alexander Kaplan

**Affiliations:** 1 Vladimir Zelman Center for Neurobiology and Brain Rehabilitation, Skolkovo Institute of Science and Technology, Moscow, Russia, 121205; 2 Baltic Center for Neurotechnology and Artificial Intelligence, Immanuel Kant Baltic Federal University, Kaliningrad, Russia, 236041; 3 Faculty of Biology, Lomonosov Moscow State University, Moscow, Russia, 119234; 4 Faculty of Mechanics and Mathematics, Lomonosov Moscow State University, Moscow, Russia, 119991; 5 Sechenov Institute of Evolutionary Physiology and Biochemistry of the Russian Academy of Sciences, Saint Petersburg, Russia, 194223

**Keywords:** EEG, event-related desynchronization, mental imagery, sensorimotor cortex, tactile imagery, tactile stimulation

## Abstract

It is well known that both hand movements and mental representations of movement lead to event-related desynchronization (ERD) of the electroencephalogram (EEG) recorded over the corresponding cortical motor areas. However, the relationship between ERD in somatosensory cortical areas and mental representations of tactile sensations is not well understood. In this study, we employed EEG recordings in healthy humans to compare the effects of real and imagined vibrotactile stimulation of the right hand. Both real and imagined sensations produced contralateral ERD patterns, particularly in the μ-band and most significantly in the C3 region. Building on these results and the previous literature, we discuss the role of tactile imagery as part of the complex body image and the potential for using EEG patterns induced by tactile imagery as control signals in brain-computer interfaces (BCIs). Combining this approach with motor imagery (MI) could improve the performance of BCIs intended for rehabilitation of sensorimotor function after stroke and neural trauma.

## Significance Statement

In this study, we address the issue of mental representations in the somatosensory domain. By assessing the dynamics of sensorimotor EEG rhythms and the distribution of topographical EEG patterns, we demonstrate that tactile imagery produces event-related desynchronization (ERD) in the contralateral EEG, even in the absence of physical stimulation. Our results clarify the neurophysiological mechanisms underlying the occurrence of ERD in the μ rhythm and its relationship to somatosensory cortical processing.

## Introduction

The capacity to form internal mental representations of physical activities, such as overt actions and their associated sensations, which is also referred to as mental imagery, is a fundamental aspect of human cognition. Mental imagery could be associated with cognitive functions, including memory, creativity, motor control, navigation, arithmetic, moral decisions, and mind wandering (Pearson, 2019). One of the most studied forms of mental imagery is motor imagery (MI), which induces neuroplasticity changes at the cortical level and facilitates motor learning (Ruffino et al., 2017; Ladda et al., 2021). MI practice is used as supplementary training in sports (Mizuguchi et al., 2012a) and neurorehabilitation (Machado et al., 2019). Depending on the sensory modality, kinesthetic and visual types of motor imagery have been distinguished (Stevens, 2005). These two distinct strategies have specific effects on corticospinal excitability (Stinear et al., 2006), electroencephalogram (EEG) activation patterns (Neuper et al., 2005), and metabolic brain activation (Guillot et al., 2009). Studies suggest that kinesthetic motor imagery is processed by the motor circuits that overlap with the circuits activated during the preparation and execution of voluntary movements. Since imagining sensations arising from muscle contraction (muscle and skin tension, changes in joint angle) is typically a part of kinesthetic MI strategy, it is reasonable to suggest that such imagery engages somatosensory areas, as well. The somatosensory component of MI was studied experimentally (Naito et al., 2002) and analyzed theoretically (Grush, 2004). Recent studies have also shown that somatosensory inputs can facilitate cortical excitability during MI (Mizuguchi et al., 2009, 2011, 2012b; Kaneko et al., 2014; Yakovlev et al., 2019).

A transient decrease in EEG/magnetoencephalography (MEG) power in a specific frequency band (Pfurtscheller and Da Silva, 1999; Neuper et al., 2006) called event-related desynchronization (ERD) is a conventional marker of sensorimotor processing. ERD and the opposite process called event-related synchronization (ERS) represent changes in correlated activity of the underlying neuronal populations, and these changes correspond to increased and decreased cortical processing, respectively (Pfurtscheller and Da Silva, 1999). As such, ERD/ERS can be used for functional cortical mapping (Pfurtscheller, 2001; Zhao et al., 2019) or as classification features for brain computer interfaces (BCIs; Pfurtscheller and Neuper, 2006; McFarland and Wolpaw, 2017; Vasilyev et al., 2017). Sensorimotor rhythms is a type of oscillatory activity in the α (8–12 Hz) and β (13–30 Hz) ranges that occurs in cortical sensorimotor regions during voluntary movements, motor imagery, movement observation, and tactile stimulation (Hari and Salmelin, 1997). It is assumed that the sensorimotor α (also referred as to μ-rhythm) is generated in the primary somatosensory cortex, whereas sensorimotor β is generated in the primary motor cortex (Hari and Salmelin, 1997). Frolov et al. (2012) and Frolov et al. (2014) confirmed the involvement of primary somatosensory areas in μ-rhythm generation. They found that motor imagery-associated EEG activity and blood-oxygen level-dependent (BOLD) signals had the same source localized in the hand representation of the sensorimotor cortex.

While studies on MI are plentiful, fewer research has been conducted on tactile imagery (TI). Neuroimaging studies of TI are limited to the fMRI method (Yoo et al., 2003; Schmidt et al., 2014; Schmidt and Blankenburg, 2019), and there is a lack of systematic work with the EEG approach. Yoo et al. (2003) reported activation of the sensorimotor cortex caused by mental imagery of brushing stimuli. Schmidt et al. provided evidence of changes in coupling between cortical areas during TI (Schmidt et al., 2014; Schmidt and Blankenburg, 2019). Additionally, Bashford et al. (2021) observed cortical activity in human subjects imagining the sensations that were previously evoked by intracortical microstimulation (ICMS) of their somatosensory cortex using an implanted microelectrode array. Somatosensory imagery (also referred to as somatosensory attention orientation, SAO) has been proposed as a complement (Yao et al., 2017a) or alternative (Yao et al., 2018, 2022a) to MI for use in BCIs. Yao and colleagues demonstrated that SAO can be reliably decoded using a BCI paradigm and used as an independent modality. They also improved SAO classification by using congruent tactile stimulation (Yao et al., 2017b, 2022b) and showed that the sources of sensorimotor ERD are mainly located in the somatosensory cortex (Yao et al., 2022a). Overall, these studies demonstrated similarity between neural patterns exhibited during real perceptions and TI tasks, which is consistent with the results of MI studies showing that overlapping brain regions are engaged in physical movements and motor imagery.

Building on these previous results, here we further clarified the EEG correlates of TI. By analogy with the MI, we expected that TI would modulate the contralateral μ-rhythm. In agreement with this expectation, we observed ERD of the μ-rhythm during both tactile stimulation (TS) and TI, the finding that adds to the literature on mental imagery and contributes to the development of a new generation of BCI technologies.

## Materials and Methods

### Participants

Twenty healthy right-handed volunteers (age 22.5 ± 2.8 years; nine females) without a history of neurologic disorders took part in this study. All of them had no prior experience in mental imagery of the motor and tactile types. The study protocol was approved by the Lomonosov Moscow State University ethical committee and followed the Declaration of Helsinki. All participants were informed about the experimental procedures and signed an informed consent.

### Experimental procedure

The experiment duration did not exceed 90 min. During an experimental session, subjects sat in a comfortable armchair in a room with uniform lighting. Visual cues were presented on a 24-inch LCD monitor positioned at the distance of ∼1 m from the subjects’ eyes.

**Figure 1.**
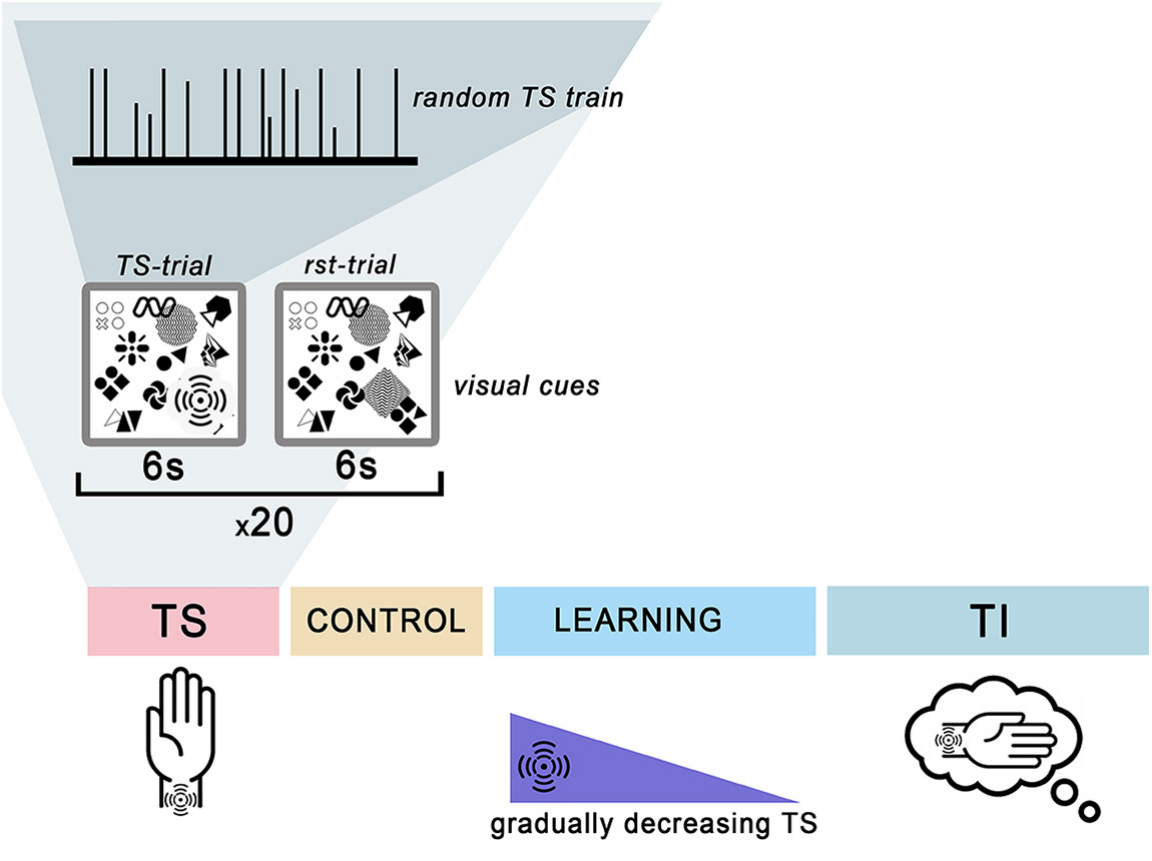
Block scheme of the experimental session. The session consisted of four consecutive conditions: tactile stimulation (TS), control, learning and tactile imagery (TI) organized in runs. Each run was a random mixture of 20 reference state (rst) trials (counting visual objects) and 20 somatosensory trials (or control with the same visual cue but no stimulation). Specifically, the TS condition had TS and rst trials, the control condition had control and rst trials, the learning condition had TS trials with decreasing stimulus amplitude and rst trials, and the TI condition had TI and rst trials. The duration of each trial was 6 s. Vibrotactile stimulation was composed of pulses of vibration of variable frequency.

An experimental session consisted of four consecutive conditions signaled by screen cues: TS, control, learning of TI, and TI. This experimental sequence is explained in Figure 1 and Table 1. Each condition comprised randomly mixed trials. The duration of each trial was 6s. The trials came in four varieties: TS, TI, control and the reference state (rst). TS and TI trials were cued by the corresponding pictograms. During TS trials, a vibrotactile stimulus was presented. TI trials required imagining being stimulated in the absence of the stimulus. During control trials, the TS pictogram was shown but no tactile stimuli were presented and TI was not required. During rst trials, participants mentally counted objects shown on the screen (circles, dots, lines, squares, etc.; see Fig. 1) while avoiding explicit eye movements. We chose to use rst-trials for consistency with the previous studies of MI where similar complex visual scenes were used as a reference state (Tangwiriyasakul et al., 2013; Vasilyev et al., 2017; Vasilyev et al., 2021). A gray screen was shown during the intertrial intervals, which lasted 300–400 ms.

**Table 1:** Experimental session composition

Condition	Runs	Trials (in one run)	Active trials, total
Tactile stimulation (TS)	1	20 TS + 20 rst	20
Control	1	20 control + 20 rst	20
Learning	2	20 TS (decreasing) + 20 rst	40
Tactile imagery (TI)	2	20 TI + 20 rst	40

As shown in Table 1, the TS condition included 20 TS trials randomly intermixed with 20 rst trials. The control condition was a mixture of 20 control trials and 20 rst trials. The learning condition included 20 TS trials intermixed with 20 rst trials. Stimulation intensity gradually decreased, and participants were required to train their TI. Finally, the TI condition was a mixture of 20 TI and 20 rst trials. In summary, participants started with experiencing vibrotactile stimulation or counting visual objects (TS condition). Next, a control was run with the same visual cues but no stimulation. In the learning condition, vibrotactile stimulation resumed, but its amplitude decreased, and participants had to perform TI. During the TI condition, participants imagined being stimulated in the absence of actual stimulation or counted visual objects, depending on the trial type.

### Selection of the site for tactile stimulation and imagery

The skin area for the application of tactile stimulation was selected based on the tests in ten subjects, which preceded the main series of experiments. We sought for the place on the arm where tactile stimulation would be well perceived without any unpleasant sensations. This perception could be then reproduced as mental imagery. The subjects did not have any prior experience in imagery, and four of them participated later in the main experimental session. Vibrotactile stimuli were delivered with vibration motors, which were placed over several locations on the right arm, including index finger, thumb, palm, and inner wrist surface. We used 20 trials per stimulation site, followed by the participants’ attempts to imagine the tactile sensation. The participants were then asked to describe their subjective experience and select the site where the stimulation-evoked sensation was the easiest to imagine.

Based on the results of these tests, the surface of the distal phalanx of the index finger and inner surface of the wrist were chosen as the most suitable places for TS and TI. Of these two locations we preferred the wrist and chose not to apply TS to the fingers for several reasons. First, finger stimulation led to long-term residual sensations that could continue into the other trials (Macefield et al., 1996; Ribot-Ciscar et al., 1996), which were also supported by the self-report from the subjects. Second, EEG modulations during finger stimulation are not hemispherically specific. According to (Vasilyev et al., 2016; Genna et al., 2017), mental imagery of finger movements, as well as tactile stimulation of the fingers, result in bilateral μ-ERD patterns whereas stimulation of more proximal parts of the arm cause predominantly contralateral ERD patterns (Le Franc et al., 2021; Li et al., 2021). Third, we wanted to avoid any potential association of TS with motor movements which could have been the case for stimulation of the finger.

### Tactile stimulation trials

We used a custom designed and computer-controlled vibrotactile stimulator based on an Arduino UNO. A flat vibratory motor (6 mm in diameter, max speed of 12 000 rpm) was placed on the inner surface of the right wrist. The stimulation continued for the entire 6-s trial duration. Variable-frequency stimulation patterns were used: stimulation was delivered as 100 ms with vibratory frequency picked from the range 3700–12,000 rpm, and the time interval between the pulses varied in the range 200–500 ms (Fig. 1). This random-frequency stimulation reduces tactile habituation and helped to avoid residual tactile sensations.

### Control trials

Since the TS and TI trials were cued with the same visual stimulus (vibration pictogram), we had to control for the possible effects of this cue. Repeated presentation of such cues leads to the development of cross-modal association (Zhou and Fuster, 2000; Bor et al., 2014). As a control, we added the condition where participants observed the same visual cues during the TS and TI trials, but no stimulation occurred and imagery was not required. Twenty of these trials were randomly intermixed with 20 rst trials (*N* = 20).

### Transition to tactile imagery

Transition to TI was conducted gradually during the learning session where 20 TS trials were intermixed with 20 rst trials. The TS amplitude gradually decreased from the parameters used in the TS session to zero. The subjects were instructed to focus attention on the skin sensations evoked by TS and to memorize them. As the TS intensity decreased, participants were asked to mentally compensate for the lack of stimulation by imagining the previously experienced sensation of stronger vibration. A similar learning strategy was previously used in MI studies (Kaplan et al., 2016; Vasilyev et al., 2017; Liburkina et al., 2018).

Following the learning session, subjects provided a verbal report regarding task difficulty; they also described the imagery vividness. An objective measure of the imagery strength was given by the μ-ERD value and topographic distribution. The imagery was considered stable if a participant reported that he/she could mentally reproduce the tactile sensations at the same place where TS was applied, the imagined sensation was not associated with the limb movements and muscle contractions, and the imagery was maintained for 6 s. The data from three participants were rejected from the analysis because one of them was unable to imagine tactile sensations and in the other two no ERD occurred during both TS and TI.

### Tactile imagery

The TI condition followed the learning condition. Here, no tactile stimuli were applied; instead, participants were instructed to imagine vibrotactile sensations. TI trials were triggered with the same visual cues as the ones used for TS trials. Participants were asked to maintain imagery of vibrotactile stimulation for the entire 6-s interval during which the cue stayed on the screen. Like the TS, control and learning conditions, rst trials constituted half of the trials in the TI condition and TS trials constituted the other half. The TI condition was run twice to a total of 40 rst trials and 40 TI trials. We controlled for the absence of muscle activity by monitoring the EMG signals from the right flexor digitorum superficialis (FDS) muscle.

### EEG and EMG recordings

We recorded 48-channel monopolar EEG data at a 500-Hz sampling rate using an NVX-52 DC amplifier (MCS). Passive Ag/Cl sensors were placed in accordance with the 10/10 international montage system. The TP10 electrode was used as the reference. The skin-electrode impedance for each of the electrodes did not exceed 20 kΩ. Bandpass filtering was conducted in the range 0.1–75 Hz using a FIR filter. An additional filtering was conducted using a 50-Hz Notch filter. Raw data collection was conducted using the NeoRec software (MCS) synced with the stimulus presentation environment. Stimulus presentation was conducted via a self-written code based on the PsychoPy python-module (Peirce, 2007). We also used one bipolar lead to record EMG from the FDS muscle.

### Signal preprocessing

Raw EEG recordings were re-referenced using the common average reference (CAR), which also has spatial filtering effects (McFarland et al., 1997). Next, the signals were bandpass filtered in the range 1–30 Hz using a fourth-order Butterworth filter. Noisy channels were interpolated using the spherical spline method (Perrin et al., 1989). The preprocessed signal was divided into 8-s epochs (from −1 to 7 s relative to trial onset) for each trial. To assess the time-frequency dynamics of EEG oscillations amplitude, the Morlet wavelet transform with variable number of cycles was applied for all the extracted epochs. The frequencies of the wavelets ranged from 6 to 30 Hz with 0.4-Hz step. The full width at half maximum (FWHM) was equal to 180 ms corresponding to a spectral FWHM of 6 Hz. Then the time-frequency matrices with Morlet coefficients were converted to ERD values in percent with Equation 1, where Morlet coefficients for each timestamp within the trial were used instead of median power spectral dencity (PSD) value.

To plot the topographic distribution of ERD/S in each experimental condition, the power spectral density was calculated with the Welch method (Welch, 1967) for the epochs from 1 to 6 s relative to trial onset. The subject-specific spectral peak was detected semi-automatically (i.e., under visual inspection) in the range of 8–15 Hz. This peak was consistent across different scalp locations and stable over time. Next, a frequency interval of ±1.5 Hz around the peak frequency in the resting state was selected and used for ERD calculation for each recording channel (Eq. 1):

(1) $$ERD=\frac{{\text{PSD}}_{smr}-{\text{PSD}}_{rst}}{{\text{PSD}}_{rst}}*100\%.$$

Where PSD*_smr_* is the median spectral power across the epochs of the TS, TI or control trials in the range 1–6 s and subject-specific frequency subrange for each channel position. PSD*_rst_* defines the spectral power calculated for trials corresponding to the resting state.

### Statistical analysis

To determine significant changes in the time-frequency dynamics of oscillatory activity and spatial distribution of the ERD/S values in all the experimental conditions (TS, TI, and control), the nonparametric cluster-level paired *t* test with 10.000 permutations was used (Maris and Oostenveld, 2007). This procedure is assumption free and is widely used for multichannel data and time-frequency analysis to avoid the multiple comparisons problem. The Friedman’s test and a Wilcoxon signed-rank test as the *post hoc* statistical test were performed to determine the statistically significant differences in total ERD/S value within the full trial in TS, TI, and control conditions, as well. The Bonferroni correction was applied for the number of tested hypotheses (adjusted to *p* < 0.003 for 15 comparisons). Statistical analysis was performed via the SciPy module v.1.4.1 (Virtanen et al., 2020). For signal processing and permutation methods, the MNE-Python v.0.23 was used (Gramfort et al., 2013). For data visualization, we used the Matplotlib graphics environment (Hunter, 2007).

## Results

### Time frequency EEG dynamics during tactile stimulation and imagery

**Figure 2.**
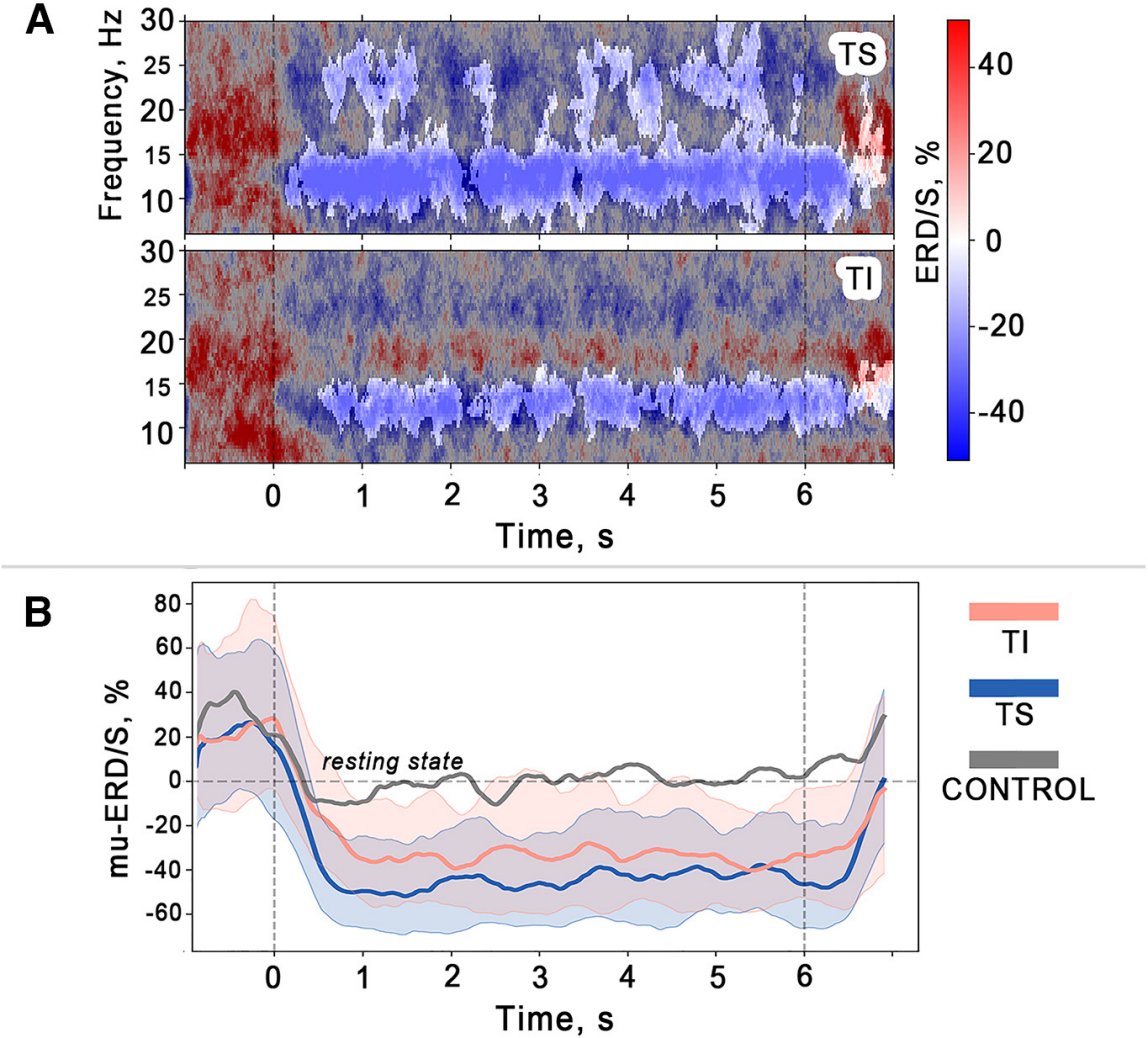
***A***, Grand median (*N* = 17) time–frequency event related desynchronization/synchronization (ERD/S) distribution in the C3 channel during the tactile stimulation (TS), tactile imagery (TI). Blue shapes correspond to the level of desynchronization (ERD), red shapes correspond to synchronization (ERS). The gray mask indicates insignificant differences (*p* > 0.003, nonparametric cluster-level paired *t* test, *p*-level adjusted by Bonferroni correction). ***B***, Grand median of the ERD/S time courses during tactile stimulation (TS), tactile imagery (TI) and control state. Color lines represent median values (median values were calculated within each subject over all trials and individual frequency ranges where ERD was detected, and then a grand median value was obtained). Color shapes show the corresponding 25th and 75th percentiles. The vertical dashed lines represent the time limit of the 6-s trial, while the horizontal line indicates the resting state.

We started with the analysis of changes in EEG rhythms for the TS and TI trials. The Wavelet-Morlet transform was applied to the signal from channel C3 (i.e., the sensorimotor area contralateral to the stimulated hand), followed by an analysis of the event-related time-frequency perturbations (TFP). We used the C3 channel for the final analysis and visualization for all participants because for all of them the highest ERD response occurred for this channel. The choice of С3 was confirmed by statistical permutation tests results. With the Morlet transformation we obtained the time-frequency representation matrices for the TS, TI, and control conditions. Then, we baselined them to the resting state (data from rst trials), calculated the median over the group and then visualized. Figure 2 shows that the group-median temporal spectral dynamics were similar during the TS and TI trials: the μ-rhythm amplitude gradually decreases relative to resting state and remains at the reduced level until the end of the trial (Fig. 2*B*). Then, it returned to the baseline. To discover significant differences between the somatosensory conditions (TS and TI) and the control state, we performed intrasubject subtraction of the time-frequency matrix corresponding to the control condition from the time-frequency matrices corresponding to TS and TI conditions. The nonparametric cluster-level paired *t* test was used to compare the obtained difference matrices with zero. We also used a similar procedure to perform comparison between the TS and TI conditions. Both the tactile stimulation and tactile imagery were characterized by a decrease across the α and β band power compared with the control condition. However, only the the decrease in the μ-rhythm power was significant for the TI-condition when it was compared with the control state (e.g., the μ-ERD clusters that persists for the entire trial duration in Fig. 2*A*), whereas a significant β-ERD occurred during the TS trials. No differences in TFP between the TS and TI were observed. Thus, the frequency range of the μ-rhythm response appearing was similar for TS and TI (∼8–14 Hz as can be defined in Fig. 2).

### ERD/S topographical patterns

**Figure 3.**
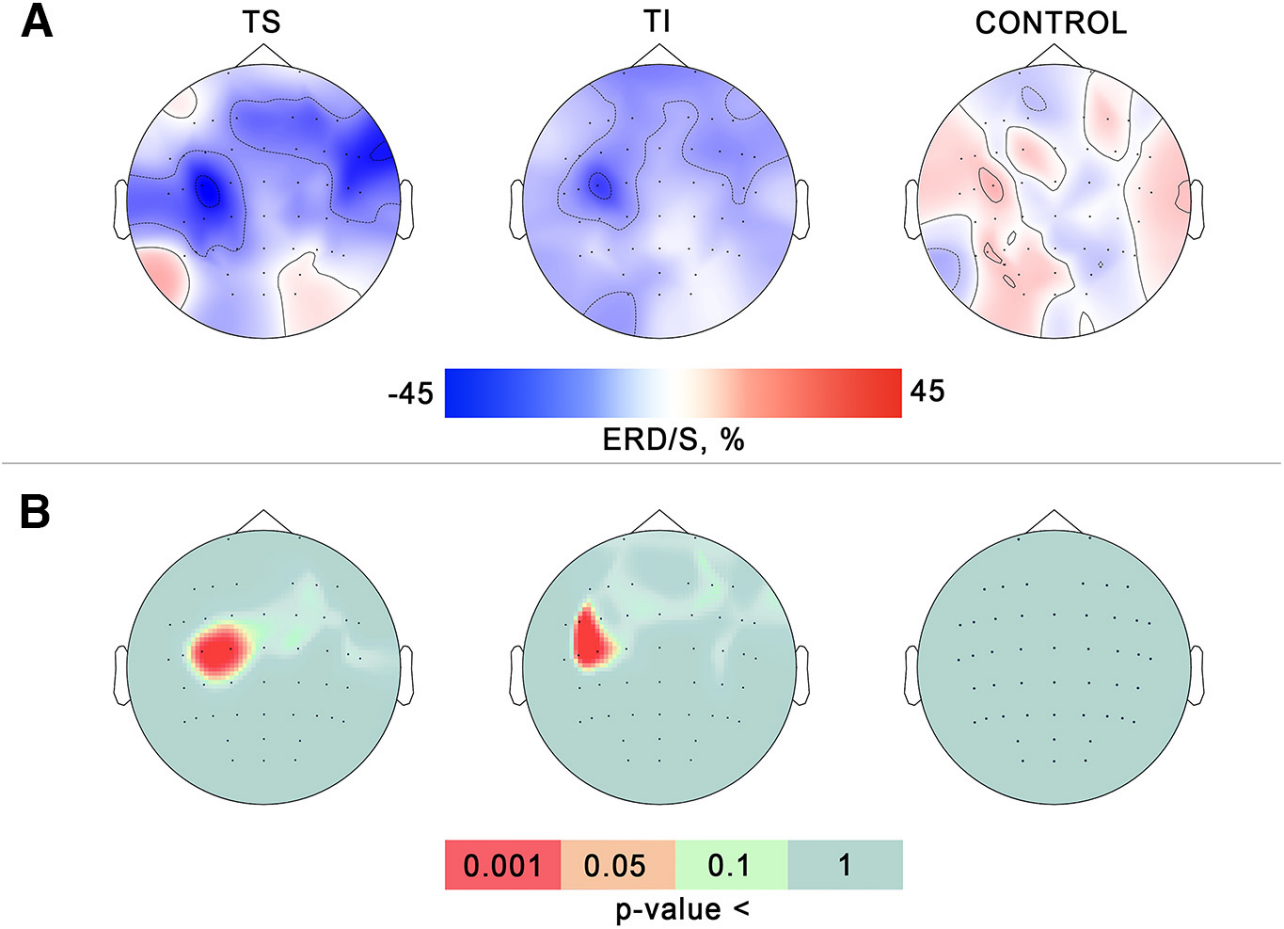
***A***, Group median event related desynchronization/synchronization (ERD/S) topomaps of sensorimotor EEG μ**-**rhythmic activity in two active conditions and the control for all subjects (*N* = 17). Blue corresponds to the level of desynchronization (ERD), red corresponds to synchronization (ERS). ***B***, The across-subjects significance maps visualized *p*-values derived from nonparametric permutation tests for all experimental conditions. Color defines the significance level. Red color marks electrodes in which there was across-subjects significance.

To determine the topographical localization of the sensorimotor response, we compared the μ*-*ERD and β*-*ERD values for each electrode position across all experimental conditions using the nonparametric permutation tests. Median (*N* = 17) topographic distribution patterns of μ*-*ERD/S for all experimental conditions are shown in Figure 3*A*. The μ*-*ERD induced by the tactile stimulation of the right-hand wrist was more prominent in the central EEG sites over the sensorimotor cortical areas, with some contralateral dominance. The TI condition had a similar μ-ERD distribution pattern. A different pattern was found for the control state.

Using a permutation *t* test relative to zero for each experimental condition, we determined the channels where significant μ-ERD occurred. The permutation *t* test revealed a significant decrease in the μ-rhythm amplitude over C3 and C1 channels for the TS condition (*t*C3 = −5.9; *p* = 0.001; *t*C1 = −6.5; *p* = 0.0007) and over FC5, C3, and C5 for the TI condition (*t*FC5 = −6.2; *p* = 0.0009; *t*C3 = −5.9; *p* = 0.0015; *t*C5 = −7.3; *p* = 0.0002). No significant μ*-*ERD topographical origins were found in the control state, that is when the tactile stimulation was not applied, and participants did not perform imagery of tactile sensations. The obtained matrices of p-values were superimposed over the scalp EEG channels positions for visualization (Fig. 3*B*). Since the TS-trials were characterized by a significant event-related desynchronization in the ∼20- to 26-Hz frequency range, we performed spatial permutation *t* test for the β*-*ERD values.

**Figure 4.**
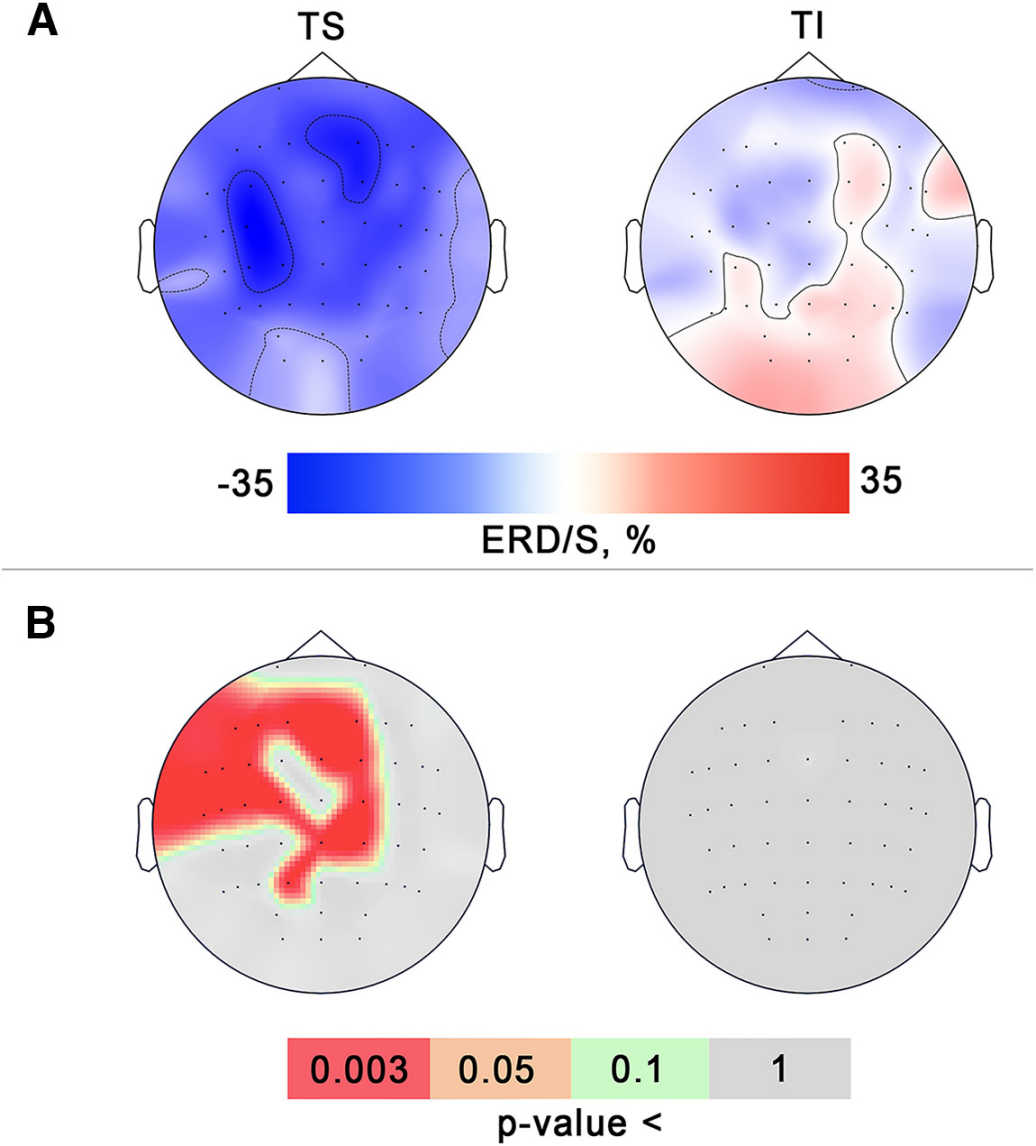
***A***, Median event related desynchronization/synchronization (ERD/S) topomaps of sensorimotor EEG β-rhythmic activity in two active conditions for all subjects (*N* = 17). Blue corresponds to the level of desynchronization (ERD), red corresponds to synchronization (ERS). ***B***, The across-subjects significance maps visualized *p*-values derived from nonparametric permutation tests for all experimental conditions. Color defines the significance level. Red color marks electrodes in which there was across-subjects significance.

Figure 4 shows that a significant decrease in β*-*amplitude induced by the tactile stimulation of the right-hand wrist occurred contralaterally, predominantly over the fronto-central EEG channels. Significant spatial clusters where β*-*ERD emerged were discovered neither in the TI condition nor in the control state.

**Figure 5.**
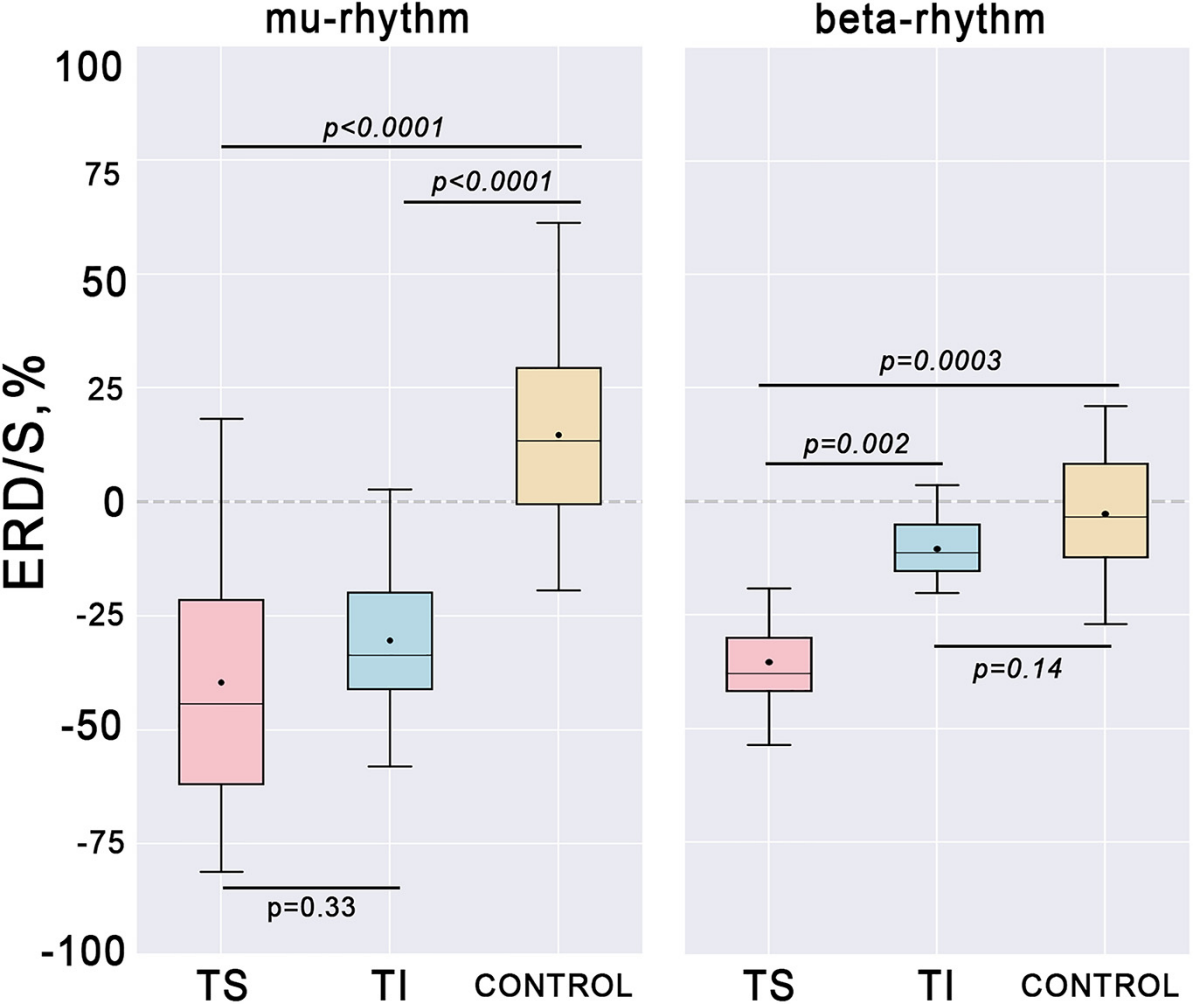
Group level (*N* = 17) ERD-values in the C3 channel for explored somatosensory conditions (tactile stimulation (TS), tactile imagery (TI), and control condition). Horizontal lines within the boxes, medians; boxes, interquartile range and [Q1 – 1.5*IQR; Q3 + 1.5*IQR] range is shown by whiskers. Circles represent mean values for the group; *p*-values are shown for the Wilcoxon signed-rank test.

To analyze the global μ*-*amplitude and β*-*amplitude decrease within the epoch duration, we calculated the median ERD value over all trials for the interval starting with the first s till the end of the trial, and over the individual frequency ranges where ERD was detected across all the participants. We rejected the first s of each active trial because of the TI onset jitter, which contributed to a gradually developing ERD (Fig. 4). The first s of the rst trials was rejected to avoid the residual effects of the tactile stimulation and imagery. There were significant differences between the experimental conditions according to Friedman’s test in μ*-*ERD value in the C3 channel (χ^2^ = 25.7; *p* = 0.000002). The paired comparisons revealed no significant difference between the ERD values in TS and TI conditions (*W* = 56; *p* = 0.33; see Fig. 5). On the other hand, as shown in Figure 5, the μ*-*desynchronization in TS and TI conditions was significantly stronger compared with the control state (*W* = 0; *p* < 0.0001). The obtained statistical data are shown in Table 2.

**Table 2:** Performed statistical analysis of sensorimotor ERD over C3 channel depending on the experimental condition

	Comparisons	Wilcoxon signed-rank test	*p*-value
μ-ERD	TS vs TI	56.0	0.33
μ-ERD	TS vs control	0.0	0.0003
μ-ERD	TI vs control	0.0	0.0003
β-ERD	TS vs TI	13.0	0.002
β-ERD	TS vs control	1.0	0.0003
β-ERD	TI vs control	46.0	0.14

A statistical analysis of the β-ERD values revealed significant differences between the three experimental conditions (χ^2^ = 15.6; *p* = 0.0004). Further analysis (Wilcoxon signed-rank tests) indicated that β-ERD was significantly stronger in the TS-condition as compared with the TI and control conditions (*W* = 13; *p* = 0.002; *W* = 1; *p* = 0.0003, respectively). The obtained statistical data are provided in Table 2.

## Discussion

In this study, we examined the changes in sensorimotor rhythms during real and imagined vibrotactile stimulation. We found that vibrotactile stimulation of the wrist as well as its imagery caused a prolonged decrease in the EEG power in the μ-range. Time-frequency analysis showed a significant μ*-*ERD in the 9–14 Hz range throughout the 6-s TS or TI trials. Additionally, TS (but not TI) caused significant β-ERD in the frequency range of 20–26 Hz. The frequency range where sensorimotor ERD appeared during TS and TI, as well as its spatial distribution, were similar to the well-known ERD patterns associated with real and imagined motor activities. This result raises the question regarding the similarities and differences in the ERD source localization for the motor versus somatosensory induced responses. We observed that cortical location of the μ-ERD evoked by TI was similar to the location previously described for MI. Although it is tempting to interpret this result as a proof of a common neural substrate for both MI and TI needed for the processing of somatosensory sensations associated with movements, EEG recordings have a limited spatial resolution, so a more accurate mapping of ERD sources should be conducted in the future for different types of mental imagery. The previous literature on MI suggests that the μ-rhythm ERD originates in the somatosensory cortex while β-ERD is mainly localized to the precentral areas (Hari and Salmelin, 1997; Frolov et al., 2012; Frolov et al., 2014). Specifically for TI, Yao et al. (2022a) suggested that ERD in the frequency range 8–26 Hz originates in sensorimotor cortex. They, however used Colin27 template for source localization, which potentially could have resulted in an overlap between the α-sources and β-sources.

Yao et al. (2018, 2022a) recently proposed using TS and TI-evoked ERD for controlling a BCI. They used vibratory stimulation that was like ours, and they also stimulated the wrist. While their work was mainly focused on constructing a BCI, we looked at the details of ERD associated with TI and added several new methodologies. First, we performed a systematic learning session where subjects familiarized themselves with the vibratory induced perceptions. Subjects were naive to mental imagery tasks in our study. Additionally, participants’ imagery trials were not mixed with any kind of physical stimulation in our study, which is different from the work of Yao and colleagues who presented 200-ms pulse of vibration before each trial to precondition mental imagery, which could have added bottom-up effects to the ERD.

Second, we used a mixed design with an equal number of somatosensory and reference trials, which also had equal durations. In the studies of Yao and colleagues, a short period before the start of the trial (from −2 to −1.2 s) was used as a reference for TI-related effects. However, such an individual reference for each trial is prone to drifts related to changes in the participant’s physiological state during the experimental session. Our calculation of the reference was different from this method. We calculated the reference as the median oscillation power from all rst trials. This approach increased the statistical power of our findings and made the results more reliable. Additionally, using a visual scene as a resting state switched attention from the somatosensory task and reduced occipital α activity during the resting state, which is more robust for ERD estimation during imagery tasks (Vasilyev et al., 2017; Vasilyev et al., 2021). For the group-level analysis, we used the median calculation and nonparametric statistics to obtain more robust and reliable results for a relatively small sample size (*N* = 17).

Third, in the studies of Yao and colleagues, ERD was derived from a wide frequency band that comprised both α and β activity (8–26 Hz), whereas we analyzed ERDs for the μ-bands and β-bands separately whose cortical sources are different. The effects of TI were different for these bands. Additionally, we analyzed individual μ-rhythm ranges for each participant to account for the intersubject differences. Overall, our results are consistent with the results of Yao et al. (2018, 2022a) and complement their work.

### Sensorimotor ERD during tactile stimulation and tactile imagery

Electrical stimulation of the median nerve is a widely used method for exploring cortical effects of afferent stimulation. Many studies showed that median nerve stimulation leads to prominent contralateral ERD of the sensorimotor EEG rhythms (Salenius et al., 1997; Nikouline et al., 2000). Similar results were observed in the studies with cutaneous vibro-tactile stimulation (Cheyne et al., 2003; Vasilyev et al., 2017). Here, we used vibrotactile stimulation of the skin area on the right wrist. We expected to obtain results consistent with the results of aforementioned studies. Such effects of afferent stimulation suggest a general principle for the relationship between α-like rhythms and sensory events: before the presentation of somatosensory stimuli, synchronized α activity is observed, and the stimuli result in α-desynchronization, possibly because of disinhibition of thalamocortical circuits (Pineda, 2005).

In addition to μ-ERD, tactile stimulation led to desynchronization in the β-frequency range. Yet, this effect was not observed for TI (i.e., was statistically insignificant). β-Oscillations have been linked to the activity of the primary motor cortex activity as it is well known that somatosensory inputs modulate β-oscillations because of motor-cortical mechanisms (Gaetz and Cheyne, 2006; Illman et al., 2021, 2022). These effects are driven by the ascending projections to M1 (Home and Tracey, 1979; Lemon, 1981) and by corticocortical connections with the somatosensory cortex (Jones, 1983). We suggest several reasons for the absence of β-ERD during TI. The first possibility is that TI is maintained by sustained activity of the primary and secondary somatosensory areas without the engagement of the motor cortex (Zhou and Fuster, 1997; Yoo et al., 2003). Indeed, participants did not imagine any movements in our experiments, so the activation of motor circuits could be weak. The second possibility is that the motor cortex responded during TS but not TI is that application of vibration to FDS generated a strong input to the motor cortex because of the activation of muscle spindle afferents (Edin, 1990). Stimulation of muscle spindles could evoke kinesthetic illusions, but this was not the case in our experiments particularly because the participants were instructed not to imagine limb movements. The third possibility is a relatively low level of alertness during TI compared with TS. Thus, Illman et al. (2021) suggested that alertness level has an effect on the sensorimotor β-desynchronization during tactile stimulation. Yet, the effects they observed were not statistically significant at the group level.

Notably, despite the absence of statistically significant β-ERD during TI, we observed a slight decrease in β power in this condition. This matches the general mental imagery model, which considers mental imagery as a reverse form of perception process with weaker cortical manifestations (Munzert et al., 2009; Dentico et al., 2014; Pearson et al., 2015). According to this model, mental imagery is based on the processing of information retrieved from memory by cortical areas. Following this idea, we propose that tactile stimulation can lead to memory formation, and this memory could be brought back to somatosensory areas when the previously experienced sensation is reproduced by imagining. Our results and the proposed interpretation agree with the simulation theory of motor imagery formulated by Jeannerod (2001).

### Tactile imagery perspectives

The tactile sense is one component of the complex motor image (Kilteni et al., 2018), in fact, the tactile imagery is a part of the motor imagery, which includes kinesthetic sensations and visual image as well. Motor imagery is a widely used mental technique in sport to improve performance and motor imagery based BCIs are promising tools for the poststroke motor recovery. However poststroke patients often have difficulties in motor imagery learning. Tactile imagery similarly to motor imagery can be proposed as a BCI control paradigm based on μ-ERD detection as well (Yao et al., 2018, 2022a; Yakovlev et al., 2022). We suppose that somatosensory mental images are easier to perform, and the learning of tactile imagery is more regulated and understandable compared with motor imagery, thus TI-based BCIs could be more useful and lower barriers to entry compared with MI-BCIs. There are a lot of studies showing the usefulness of tactile feedback and tactile stimulation during motor imagery training and MI-BCI control (Zhang et al., 2021; Sakamaki et al., 2023; Zhong et al., 2023). We believe that the positive effect of TS on the learning on kinesthetic imagination of movements may be associated with the use of mental tactile sensations (i.e., participants can actually use the tactile imagery to increase the vividness of motor image and induсe the sensorimotor rhythms depression more effectively).

### Significance of the obtained results

Although several previous studies have already examined the effects of TI, our results are novel pertaining to EEG correlates of TI following learning to imagine vibrotactile stimulation. We observed μ-rhythm desynchronization in the contralateral hemisphere during both TS and TI and β-desynchronization during TS only, which we interpreted as activation of both somatosensory and motor cortices during TS and activation of somatosensory cortex only during TI. Our results generally match the plentiful previous results on motor imagery (Pfurtscheller and Neuper, 1997; McFarland et al., 2000) assessed with EEG technique. The results we obtained also match the fMRI studies where tactile imagery was found to increase somatosensory cortical activity and enhance functional connectivity between the prefrontal and somatosensory cortical areas (Schmidt et al., 2014; Schmidt and Blankenburg, 2019). Our results also supplement recent EEG work on somatosensory imagery (Yao et al., 2018, 2022a). The other study that our results agree with is the work (Bashford et al., 2021) where microelectrode techniques were used to localize cortical regions involved in the tactile image formation. Our work is also relevant to the research of spinal circuits where tactile and kinesthetic types of processing lead to increased spinal excitability (Bunno, 2019). Overall, our work adds to the existing tactile imagery literature and is also relevant to the field of brain-computer interfaces, where tactile imagery could be used to obtain EEG responses desired for communication, control of external devices and rehabilitation purposes (Yao et al., 2017a,b, 2018, 2022a,b).

### Limitations

The results of the current study provide convincing evidence that TI leads to contralateral μ-rhythm ERD, and this effect is similar to the one evoked by the real vibrotactile stimulation applied to the wrist. However it is important to mention several limitations to our research. First, EEG recordings allowed us to observe ERD/S responses to sensorimotor stimuli, but we were unable to precisely localize the source of ERD using this method. High-density EEG recordings combined with inverse solution computing (e.g., MNE, sLORETA) could provide more detailed information about the sources of imagery-related activity and could help to better compare the effects of tactile stimulation, tactile imagery and motor imagery. Other neurophysiological techniques besides EEG, like transcranial magnetic stimulation (TMS), MEG, functional near-infrared spectroscopy (fNIRS), could be useful for this purpose, as well.

We used a visual scene as a reference state and also used it in the control condition to exclude the conditioning effect of the visual pictogram used for the TS/TI tasks. The obtained results suggest that sensorimotor rhythm modulation was the specific effect of TI. Yet it could be useful to have an additional control for tactile attention in future studies. There could be a somatosensory attention condition, where the subject’s attention is drawn to the stimulation site, but without performing imagery.

Another limitation in this study is participants’ age range, since most of the subjects were relatively young (from 19 to 30 years old). It is important to complement our findings with a broader-age group in the future. Since a decline in sensorimotor functions is typically observed with aging, it would be of interest to study the effect of age on the EEG patterns.

In conclusion, based on EEG recordings conducted during tactile imagery, we conclude that the effects of TI (namely, μ-rhythm event-related desynchronization) are similar to the effects of MI. Such mutuality of motor and somatosensory cortical processing, at least at the level of EEG, could indicate that the “imagery circuit” of the brain is not strictly localized. Yet, by changing imagery requirements it is possible to change activity distribution in somatosensory versus motor areas. Considering the sensory origin of the α rhythms in general, α-ERD during TI is yet another phenomenon where somatosensory processing is affected by thought. As such, it could be used in BCI design where thought needs to be communicated from the brain to the external devices.

## Synthesis

Reviewing Editor: Frederike Beyer, Queen Mary University of London Decisions are customarily a result of the Reviewing Editor and the peer reviewers coming together and discussing their recommendations until a consensus is reached. When revisions are invited, a fact-based synthesis statement explaining their decision and outlining what is needed to prepare a revision will be listed below. The following reviewer(s) agreed to reveal their identity: NONE.

Dear Dr. Yakolev,

Thank you for submitting your manuscript to eNeuro. I apologize for the time it took to get back to you with a decision, but we have now secured two reviews from experts in the field. Both reviewers highlight the quality and interest of your work, but raise several issues that should be addressed in any potential revision. key points include a more thorough discussion of existing studies on this topic, more details on some methodological aspects, and checking the consistency and accuracy of the writing. Please also ensure that your revised version includes a dedicated ‘participants’ section in the methods section, detailing the number and demographics of participants, as well as any exclusion criteria and the number of participants excluded from data analysis. The detailed comments of both reviewers are given below. Please ensure all comments are addressed in your revised submission.

Best wishes,

Frederike Beyer

Reviewer #1 comments

In this study, the authors aim to analyze the brain oscillatory activity (i.e. Event-Related Desynchronization (ERD)) induced by tactile imagery and its relationship with that induced by real tactile stimuli. In the experiment, the subjects first performed the tactile stimulation trials, then carried out the learning stage to learn the tactile imagery, and finally performed the tactile imagery trials. The experimental design is interesting, however, the existing literature hasn‘t yet been well-referenced and the stated novelty is disputable. I have the following comments:

Major Comments:

1. Considering that previous studies have drawn similar conclusions, I think the innovation of this work is limited. Please refer to the following studies, and discuss the improvement compared with them. More work is needed in the introduction and discussion parts to refer to the related works: The tactile imagery-based BCI system was first proposed in [1] [2][3]. It was approved that the tactile stimuli can help to calibrate the tactile imagery-based BCI [3][4].

2. In terms of experimental design, please explain why the number of active trials under different conditions is different. Will this have a bad effect on the experimental results? And please discuss why the rst trials were necessary.

3. In the Signal Preprocessing section, the subject-specific peak frequency was selected by visual inspection at each channel position. Are ERD peak frequencies different at different locations within one subject?

4. In the Significance of the obtained results section, it seems inappropriate to say “While TI was the subject of several previous studies, here we studied its manifestation in EEG for the first time.”. Please refer to question one, and a more comprehensive related literature review should be done.

5. In lines 351∼352, the spatial distributions of TS and TI were similar to the well-known ERD patterns associated with real and imaginary motor activities. Is it possible the μ-band ERD in TS or TI is from the same source as that in motor imagery? Please discuss more the relationship among TS, TI, and motor imagery, such as the ERD components and the sources.

Minor Comments:s

1. In lines 139∼140, please add some references to “First, finger stimulation led to long-term residual sensations that could continue into the other trials.”

2. In lines 245-246, the authors described that Figure 2 was calculated by group average, which was different from the grand median in the caption of Figure 2. Does the median mean the same as the average in this article? Please ensure consistency.

3. In the Tactile imagery perspectives section, please add some references to “The tactile sense is one component of the complex motor image...”.

[1] L. Yao, X. Sheng, D. Zhang, N. Jiang, D. Farina, and X. Zhu, “A BCI System Based on Somatosensory Attentional Orientation,” IEEE Trans Neural Syst Rehabil Eng, vol. 25, no. 1, pp. 78-87, Jan. 2017, doi: 10.1109/TNSRE.2016.2572226.

[2] L. Yao, N. Mrachacz-Kersting, X. Sheng, X. Zhu, D. Farina, and N. Jiang, “A Multi-Class BCI Based on Somatosensory Imagery,” IEEE Transactions on Neural Systems and Rehabilitation Engineering, vol. 26, no. 8, pp. 1508-1515, Aug. 2018, doi: 10.1109/TNSRE.2018.2848883.

[3] Yao, Lin, Ning Jiang, Natalie Mrachacz-Kersting, Xiangyang Zhu, Dario Farina, and Yueming Wang. “Performance Variation of a Somatosensory BCI Based on Imagined Sensation: A Large Population Study.” IEEE Transactions on Neural Systems and Rehabilitation Engineering 30 (2022): 2486-2493.

[4] L. Yao, X. Sheng, N. Mrachacz-Kersting, X. Zhu, D. Farina, and N. Jiang, “Decoding Covert Somatosensory Attention by a BCI System Calibrated With Tactile Sensation,” IEEE Transactions on Biomedical Engineering, vol. 65, no. 8, pp. 1689-1695, Aug. 2018, doi: 10.1109/TBME.2017.2762461.

[5] L. Yao, N. Jiang, N. Mrachacz-Kersting, X. Zhu, D. Farina, and Y. Wang, “Reducing the Calibration Time in Somatosensory BCI by Using Tactile ERD,” IEEE Transactions on Neural Systems and Rehabilitation Engineering, vol. 30, pp. 1870-1876, 2022, doi: 10.1109/TNSRE.2022.3184402.

Reviewer #2 comments

It would make the manuscript better if there was more consistency in the writing. More particularly:

- duration: sometimes written as 5s, sometimes 5 sec and sometimes 5 sec...

- In the text, the figures are sometimes referred as Fig. and sometimes as Figure

- some abbreviations were not explained first. e.g. TMS (line 51), BOLD (line 71), rst in Table 1?

- the use of abbreviations, It should be explained first before using only the abbreviation for the rest of the manuscript. e.g.: FDS (line 179) explained later in line 198.

Table 1 and Table 2 were never mentioned in the text.

Section Materials and Methods, there was only one subsection: Experimental procedure. Is “Selection of the Site for Tactile Stimulation and Imagery) the same level as Materials and Methods? Because it appears so, although I think it should be a subsection. Same goes with “Control Trials”.

It is unclear to me how many subjects took part of any specific part of the experiment. Line 125 says there are 10 subjects. Then line 129 says that 4 of them participated in the main experimental sessions. Afterwards, in line 170, it says that data from 3 participants were rejected?

Were the subjects right-handed? It would be interesting to find out if this could factor the results.

Line 240, Wavelet-Morlet transform was applied to C3. Only C3? if yes, why? The reason was not provided here.

Figure 2A, please also provide the labels in the y-axis for TI.

Figure 3A, please also show the nose and the ears like in Figure 4.

Line 432, what does “related” mean?

Finally, it would definitely benefit the paper to be edited for English language.

## Author Response

Reviewer #1 comments

In this study, the authors aim to analyze the brain oscillatory activity (i.e. Event-Related Desynchronization (ERD)) induced by tactile imagery and its relationship with that induced by real tactile stimuli. In the experiment, the subjects first performed the tactile stimulation trials, then carried out the learning stage to learn the tactile imagery, and finally performed the tactile imagery trials. The experimental design is interesting, however, the existing literature hasn’t yet been well-referenced and the stated novelty is disputable. I have the following comments:

Major Comments:

1. Considering that previous studies have drawn similar conclusions, I think the innovation of this work is limited. Please refer to the following studies, and discuss the improvement compared with them. More work is needed in the introduction and discussion parts to refer to the related works: The tactile imagery-based BCI system was first proposed in [1] [2][3]. It was approved that the tactile stimuli can help to calibrate the tactile imagery-based BCI [3][4].

ANSWER:

Thank you for your very valuable feedback. We have implemented your suggestion to refer to related works in the introduction and discussion parts. In the revised manuscript, we have incorporated a comprehensive literature review that explains how our work extends the previous reports, including the ones you suggested that we cite. Hopefully these changes will improve the quality and impact of our manuscript, and thank you again for the suggestions regarding the literature.

2. In terms of experimental design, please explain why the number of active trials under different conditions is different. Will this have a bad effect on the experimental results? And please discuss why the rst trials were necessary.

ANSWER:

Thanks for pointing to this issue. Indeed, the tactile imagery condition had 40 trials, whereas the tactile stimulation condition had 20 trials. We ran a larger number of trials for the tactile imagery condition to ensure that we have enough data for the statistical analysis where variability of EEG patterns is increased because subjects have just acquired a new mental skill. It is important to note here that EEG patterns are not as robust during tactile imagery as they are during the presentation of real tactile stimulation. Having more data would not hurt, and the different number of trials in different conditions does not compromise the statistics because we used the median as a robust estimation of the central tendency.

Regarding the resting state trials (rst), we used a mixed design where active trials were intermixed with resting state trials in random order. This approach allowed us to assess event-related desynchronization by comparing the active condition trials and the resting state trials drawn from the same session. Thus, the rst-trials provided us with a baseline measure of mu-rhythm power during the resting state, which allowed us to compare it to the mu-power during the active trials and derive the desynchronization values.

In the revised manuscript we have discussed the advantages of using that sort of experimental design. The revised text now reads:

“Secondly, we used a mixed design with an equal number of somatosensory and reference trials, which also had equal durations. In the studies of Yao and his colleagues, a short period before the start of the trial (-2 s to -1.2 s) was used as a reference for TI-related effects. However, such an 2 individual reference for each trial is prone to drifts related to changes in the participant’s physiological state during the experimental session. Our calculation of the reference was different from this method.

We calculated the reference as the median oscillation power from all rst-trials. This approach increased the statistical power of our findings and made the results more reliable. Additionally, using a visual scene as a resting state switched attention from the somatosensory task and reduced occipital alpha activity during the resting state, which we is more robust for ERD estimation during imagery tasks (Vasilyev et al., 2017; Vasilyev et al., 2021).”

3. In the Signal Preprocessing section, the subject-specific peak frequency was selected by visual inspection at each channel position. Are ERD peak frequencies different at different locations within one subject?

ANSWER:

In our study, we utilized contralateral central channels to identify the mu-rhythm peak frequency in the resting state (rst-trials). The mu-rhythm peak frequency was consistent across different scalp locations and stable over time, which is consistent with our previous research. It’s worth noting that there was an occipital alpha rhythm present in the signal, which was most pronounced in occipital channels. This alpha rhythm had a lower peak frequency (1-1.5 Hz) for all participants, but it also remained consistent across different scalp locations. We detailed our approach in the Signal Preprocessing Section, specifying the consistency of the mu-rhythm peak frequency and how we used it to estimate ERD. The revised text now reads: “The subject-specific spectral peak was detected semi-automatically (i.e., under visual inspection) in the range of 8-15 Hz. This peak was consistent across different scalp locations and stable over time. Next, a frequency interval of {plus minus} 1.5 Hz around the peak frequency in the resting state was selected and used for ERD calculation for each recording channel”

4. In the Significance of the obtained results section, it seems inappropriate to say “While TI was the subject of several previous studies, here we studied its manifestation in EEG for the first time.”. Please refer to question one, and a more comprehensive related literature review should be done.

ANSWER:

Thanks for this point. We have revised the manuscript according to this suggestion. The revised text now reads: “Although several previous studies have already examined the effects of TI, our results are novel pertaining to EEG correlates of TI following learning to imagine vibrotactile stimulation.”

5. In lines 351∼352, the spatial distributions of TS and TI were similar to the well-known ERD patterns associated with real and imaginary motor activities. Is it possible the μ-band ERD in TS or TI is from the same source as that in motor imagery? Please discuss more the relationship among TS, TI, and motor imagery, such as the ERD components and the sources.

ANSWER:

Thanks for this excellent point. Although TI is quite distinct and different from imagining limb movements, the ERD patterns are indeed similar both spatially and spectrally. At the current stage, our EEG method does not have sufficient spatial resolution, so we will have to conduct more studies in the future comparing the effects of TI and motor imagery. The revised text now reads: ”...The frequency range where sensorimotor ERD appeared during TS and TI, as well as its spatial distribution, were similar to the well-known ERD patterns associated with real and imagined motor activities. This result raises the question regarding the similarities and differences in the ERD source localization for the motor versus somatosensory induced responses. We found that cortical location of 3 the μ-ERD evoked by TI was similar to the location previously described for MI. Although it is tempting to interpret this result as a proof of a common neural substrate for both MI and TI needed for the processing of somatosensory sensations associated with movements, EEG recordings have a limited spatial resolution, so a more accurate mapping of ERD sources should be conducted in the future for different types of mental imagery. The previous literature on MI suggests that the μ-rhythm ERD originates in the somatosensory cortex while beta-ERD is mainly localized to the precentral areas (Hari and Salmelin, 1997; Frolov et al., 2012; Frolov et al., 2014). Specifically for TI, Yao and his colleagues suggested that ERD in the frequency range 8-26 Hz originates in sensorimotor cortex (Yao et al., 2022a)...”

Minor Comments:

1. In lines 139∼140, please add some references to “First, finger stimulation led to long-term residual sensations that could continue into the other trials.”

ANSWER:

Thanks! We have revised the manuscript to include the following references that support for this statement: ● Macefield VG, Häger-Ross CK, Johansson RS. Control of grip force during restraint of an object held between finger and thumb: responses of cutaneous afferents from the digits. Exp Brain Res. 1996;110(3):467-481. ● Ribot-Ciscar, E., Roll, J. P., Tardy-Gervet, M. F., & Harlay, F. (1996). Alteration of human cutaneous afferent discharges as the result of long-lasting vibration. Journal of Applied Physiology, 80(5), 1708-1715.

The revised sentence now reads: “First, finger stimulation led to long-term residual sensations that could continue into the other trials (Macefield et al., 1996; Ribot-Ciscar et al., 1996), which were also supported by the self-report from the subjects.”

2. In lines 245-246, the authors described that Figure 2 was calculated by group average, which was different from the grand median in the caption of Figure 2. Does the median mean the same as the average in this article? Please ensure consistency.

ANSWER:

Thank you for bringing this to our attention. Sorry for the confusion in our description of Figure

2. We actually used the median as the measure of central tendency, not the group mean. We have revised the manuscript to clarify this. The revised text now reads: “Figure 2 shows that the group-median temporal spectral dynamics were similar during the TS and TI trials: the μ-rhythm amplitude gradually decreases relative to resting state and remains at the reduced level until the end of the trial (Fig. 2).”

3. In the Tactile imagery perspectives section, please add some references to “The tactile sense is one component of the complex motor image...”.

ANSWER:

4

Done. We have added the following reference to substantiate the statement that tactile sensations (skin stretching, pressures, tingling etc.) are a component of motor imagery: ● Kilteni, K., Andersson, B. J., Houborg, C., & Ehrsson, H. H. (2018). Motor imagery involves predicting the sensory consequences of the imagined movement. Nature communications, 9(1), 1617.

Reviewer #2 comments

It would make the manuscript better if there was more consistency in the writing. More particularly: - duration: sometimes written as 5s, sometimes 5 sec and sometimes 5 sec...

ANSWER:

Thanks for this point. We have now changed “sec” to “s” throughout the manuscript to maintain consistency.

- In the text, the figures are sometimes referred as Fig. and sometimes as Figure

ANSWER:

Done. We now use “Figure” in figure legends and “Fig.” in the text. This is how the latest articles published in eNeuro are formatted. - some abbreviations were not explained first. e.g. TMS (line 51), BOLD (line 71), rst in Table 1?

ANSWER:

We have double-checked the abbreviations and unified them by providing an explanation each time they first appear in the text. - the use of abbreviations, It should be explained first before using only the abbreviation for the rest of the manuscript. e.g.: FDS (line 179) explained later in line 198.

ANSWER:

We have corrected this. Table 1 and Table 2 were never mentioned in the text.

ANSWER:

In the revised version of the manuscript, we have added references to these tables in the appropriate sections of the text. Section Materials and Methods, there was only one subsection: Experimental procedure. Is “Selection of the Site for Tactile Stimulation and Imagery) the same level as Materials and Methods? Because it appears so, although I think it should be a subsection. Same goes with “Control Trials”.

ANSWER:

Thank you for these suggestions. We’ve corrected the section/subsection structure.

It is unclear to me how many subjects took part of any specific part of the experiment. Line 125 says there are 10 subjects. Then line 129 says that 4 of them participated in the main experimental sessions. Afterwards, in line 170, it says that data from 3 participants were rejected? 5

ANSWER:

Yes, the reason for this misunderstanding is the section “participants” removed due to the journal rules. Thanks for this comment. We have clarified in the revised manuscript that we had 10 participants in the pilot/technical session where we conducted initial tests of vibrostimulation parameters (line 125). Of these ten, 4 subjects participated in the main experimental session, which consisted of a total of 20 subjects. To complicate this a little bit, 1 subject of the final 20 failed in being able to perform TI and additional 2 subjects did not have the mu-rhythm. This brought the number of subjects to 17 finally used in analysis and visualization. The revised text now reads: “Twenty healthy right-handed volunteers (age 22.5 {plus minus} 2.8 years; 9 females) without a history of neurological disorders took part in this study. All of them had no prior experience in mental imagery of the motor and tactile types.” Were the subjects right-handed? It would be interesting to find out if this could factor the results.

ANSWER:

Thank you for this excellent point. Yes, all the subjects in our study were right-handed. We recognize that hand dominance may be a relevant factor and, therefore, decided to include only right-handed participants. We agree that investigating the impact of hand dominance on tactile imagery and its correlates could be valuable for future research. Line 240, Wavelet-Morlet transform was applied to C3. Only C3? If yes, why? The reason was not provided here.

ANSWER:

Thank you for bringing this to our attention. Yes, only C3 was used in this specific analysis because this was the channel where the TI-related effects were most prominent. So we updated the text with the next motivation: “We used the C3 channel for the final analysis and visualization for all participants because for all of them the highest ERD response occurred for this channel. The choice of C3 was confirmed by statistical permutation tests results.” Figure 2A, please also provide the labels in the y-axis for TI.

ANSWER:

Thank you for bringing this to our attention. We have included the labels for the y-axis of TI in Figure 2A as you requested. Figure 3A, please also show the nose and the ears like in Figure 4.

ANSWER:

Thank you for your comment regarding Figure 2A. We have updated the plot to include the ears and noses, as requested. Now, the format of all topographic maps is unified to ensure consistency. Line 432, what does “related” mean?

ANSWER:

Thank you for bringing this to our attention. We have updated the sentence to correct this typo. The revised sentence now reads: “EEG recordings allowed us to observe ERD/S responses to sensorimotor stimuli, but we were unable to precisely localize the source of ERD using this method.” 6 Finally, it would definitely benefit the paper to be edited for English language.

ANSWER:

Thanks for this suggestion. We have proofread the manuscript for the English language.
